# Treatment of the Acute Alcohol Withdrawal State: A Comparison of Four Drugs

**Published:** 1995

**Authors:** Edward M. Sellers

**Affiliations:** Edward M. Sellers, M.D., Ph.D., is a professor of pharmacology, medicine, and psychiatry, University of Toronto, and senior scientist at the Addiction Research Foundation, Toronto, Ontario, Canada

**Keywords:** AOD withdrawal syndrome, drug therapy, drug efficacy, comparative study, chlordiazepoxide, thiamine, histamine antagonists, tranquilizing agents, delirium tremens, AOD-related (AODR) seizure, diagnostic criteria

**Figure:**
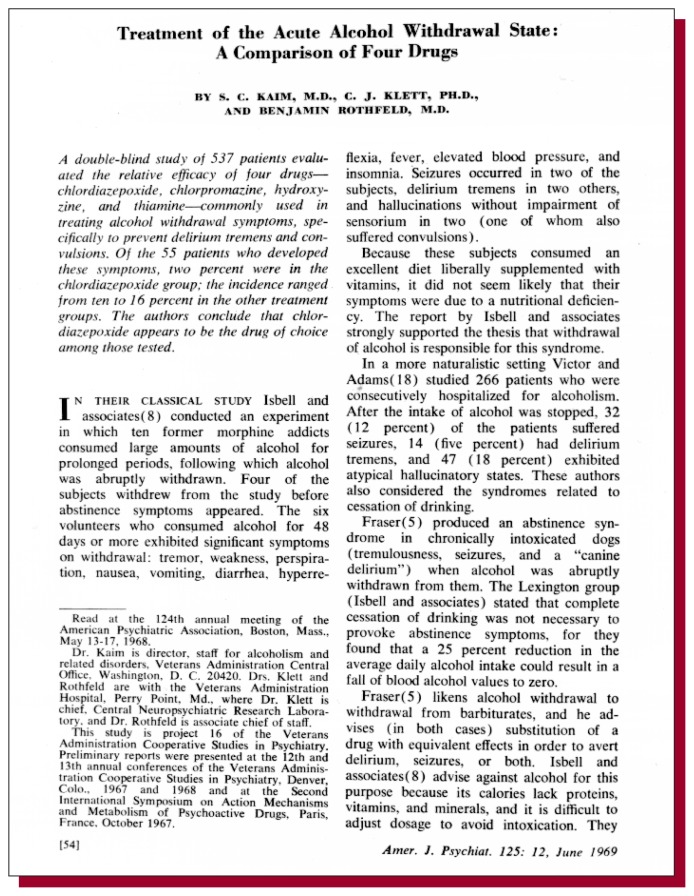
Kaim, S.C.; Klett, C.J.; and Rothfeld, B. Treatment of acute alcohol withdrawal state: A comparison of four drugs. *American Journal of Psychiatry* 125(12):1640–1646, 1969.

The seriousness of alcohol withdrawal and its widespread occurrence in the 166 Veterans Administration hospitals in the 1960’s was the principal reason this pivotal study was conducted. The important work by Kaim and colleagues has endured over the years, because alcohol withdrawal still is common, potentially life threatening, and often not recognized and treated promptly and effectively.

Kaim and colleagues studied the efficacy of four drugs—chlordiazepoxide (a benzodiazepine), chlorpromazine (a neuroleptic or antipsychotic agent), hydroxyzine (a sedating antihistamine), and thiamine (a vitamin)—commonly used at that time to treat alcohol withdrawal symptoms, specifically the more serious symptoms of delirium tremens and convulsions. These researchers’ results help establish the benzodiazepines as drugs of choice in treating alcohol withdrawal. To fully understand the context in which Kaim and colleagues conducted their study, it is useful to consider the state of pharmacological treatment of withdrawal at that time.

During the 1950’s and early to mid-1960’s, several initial studies suggested that promazine and chlorpromazine (both antipsychotic medications) were effective in treating alcohol withdrawal. Later studies did not confirm these findings. Moreover, several subsequent studies suggested that the incidence of serious withdrawal symptoms, such as seizures and delirium, might actually be higher after administration of these two agents (Sereny and Kalant 1965). Pharmacologically, based on today’s knowledge, complications such as confusion and delirium would be expected. These tranquilizing agents decrease the seizure threshold by making the brain more susceptible to spontaneous cellular electrical activity. The agents also have properties (i.e., anticholinergic) that disrupt cellular communication, resulting in changes in body function (e.g. gastrointestinal disorders).

Chlordiazepoxide was another medication studied by Kaim and colleagues in their article. Chlordiazepoxide was marketed in 1961. It is interesting to note that in that same year, Kaim and Rostenstein (1961) opined that based on clinical evidence,

Librium [chlordiazepoxide], in higher dosage of 200 to 300 mg, daily, brings prompt and gratifying control of both the psychotic and convulsive phenomena without the toxicity experienced with the use of phenothiazines, reserpine, or even the barbiturates (see Kaim et al. 1969, p. 1641).

The research evidence to support this statement was first presented orally by the authors in 1967 and then published in this 1969 report. Presumably, these early clinical impressions provided the stimulus for Kaim and colleagues’ more formal study on the four agents.

Another agent examined by Kaim and colleagues was thiamine. It may seem curious now that thiamine, a vitamin, was included as a test drug in this study. However, in the 1960’s, there still was debate as to the contribution of the combination of alcohol and poor nutrition to withdrawal state. Because many alcoholics do not eat a well-balanced diet, it was believed that nutritional deficiencies, such as thiamine, might be related to the severity of withdrawal and its complications. It was only in 1956 that Isbell first demonstrated that abrupt discontinuation of alcohol administration in well-nourished individuals still was followed by alcohol withdrawal and seizures (Isbell et al. 1955).

In the 1960’s, controversy also existed as to whether early treatment of alcohol withdrawal symptoms would prevent the progression to delirium tremens (Sellers and Kalant 1976). In their article, Kaim and colleagues suggested that early treatment could indeed halt the progression of the withdrawal state. Similarly, the etiology of seizures during withdrawal was uncertain (i.e., could seizures be prevented?). Kaim and coworkers found that chlordiazepoxide decreased the risk of seizures (compared with placebo) and chlorpromazine increased this risk. Based on these findings, the researchers deduced that the seizures that developed during alcohol withdrawal resulted from a general neuroadaptive change in the brain caused by the withdrawal of alcohol. Such changes could be modified by pharmacological factors and varied among individual patients.

Several important aspects have continued to set Kaim and colleagues’ work apart from that of other researchers. For example:

- The size of the study was quite large (557 patients)—no study, before or after, has been larger.
- The study was conducted in “real life” clinical care settings across the United States.
- A significant number of patients in the study who received only a placebo (nonactive medication) had positive treatment outcomes. Such successful outcomes demonstrated what a powerful treatment simple supportive care could be in some patients. Indeed, Sellers and colleagues (1983), among others, confirmed a high-placebo response in terms of subjective symptoms (e.g., anxiety) and nonserious physical signs (e.g., tremor). However, the placebo was not effective in decreasing the risk of serious complications (e.g., seizures, delirium).
- The apparent progression of alcohol withdrawal to the more serious delirium tremens was prevented by chlordiazepoxide. Incidentally, it is interesting to speculate that the results of this study probably understate the efficacy potential of chlordiazepoxide because the patients received only 50 mg every 6 hours, administered intramuscularly and orally. A more flexible dosing schedule with higher doses might well have shown better efficacy. Furthermore, chlordiazepoxide injections administered intramuscularly probably resulted in rather low and initially inconsistent levels of the drug in the blood.
- As mentioned earlier, chlorpromazine increased the risk of seizures during alcohol withdrawal. According to Sellers and coworkers (1983), treatment of alcohol withdrawal syndrome with a benzodiazepine is rarely associated with seizures.
- Kaim and colleagues used appropriate clinical criteria to identify and diagnose alcohol withdrawal (i.e., gastrointestinal distress, sweating and flushing [increased autonomic activity], insomnia, tremor, irritability, apprehension, depression, and clouded sensory perception or confusion). Since the 1960’s, quantitative refinements have been made in defining these criteria more precisely (e.g., *the Diagnostic and Statistical Manual of Mental Disorders, Third Edition, Revised* and the manual’s fourth edition); still the clinical criteria established by Kaim and colleagues have been largely validated (Sellers et al. 1991). However, in the study by Kaim and colleagues, a graded measure of alcohol withdrawal severity was not used; consequently, differences in the patient’s time of response to each drug were not seen, and the results of the study probably present a conservative estimate of the differences among the drugs.

If an experiment similar to the one conducted by Kaim and colleagues was performed today, the study would likely include a more detailed characterization of the patients; the use of a validated and standardized measure of alcohol withdrawal, such as the revised Clinical Institute Withdrawal Assessment of Alcohol scale (CIWA–Ar) (Sullivan et al. 1989); a data presentation to show the patient’s time of response to each drug; statistical analysis to determine the likelihood that the patient would experience complications or that the patient would have a successful treatment outcome; and the use of a strategy that allows researchers to administer more medication to patients earlier in the treatment (Sellers et al. 1983). Finally, considering what is known about thiamine today, it is unlikely that a 1995 trial would include this vitamin. Even the use of a placebo control group would be controversial because of the substantial risk that nondrug-treated patients might develop serious complications.

Apart from the general lessons imparted by the work of Kaim and colleagues and by others in the field, it is likely that this seminal article had still another major impact on the treatment of patients. It created within the Veterans Administration system an increased awareness of, and interest in, treating alcohol withdrawal effectively. From a broader perspective, benzodiazepines have continued to be the drugs of choice for alcohol withdrawal since shortly after the publication of the seminal paper (Sellers and Kalant 1976). Although refinements have been made in measurement, dose schedules, trial designs, and data analysis, the clinical trial results of Kaim and colleagues in this seminal article have been confirmed repeatedly in clinical practice since 1969. Furthermore, many of the observations have been incorporated into “clinical pearls” (e.g., always treat a patient experiencing alcohol withdrawal with a benzodiazepine first before you give a neuroleptic if the patient is hallucinating). We now know that some patients may need a drug such as haloperidol (a drug similar to chlorpromazine) in addition to benzodiazepine to fully treat their withdrawal.
